# New Approaches to Continuing Medical Education: a QStream (spaced education) Program for Research Translation in Ovarian Cancer

**DOI:** 10.1007/s13187-015-0944-7

**Published:** 2015-11-15

**Authors:** Tracy Robinson, Anna Janssen, Judy Kirk, Anna DeFazio, Annabel Goodwin, Kathy Tucker, Timothy Shaw

**Affiliations:** 1 Faculty of Nursing and Midwifery, University of Canberra, Sydney, Australia; 2 Sydney West Translational Cancer Research Centre, Crown Princess Mary Cancer Centre, Westmead Hospital, Sydney, Australia; 3 Research in Implementation Science and eHealth, Faculty of Health Sciences, University of Sydney, Sydney, Australia; 4 Familial Cancer Services, Westmead Hospital, Sydney, Australia; 5 Royal Prince Alfred Hospital, Sydney, Australia; 6 Sydney Catalyst Translational Cancer Research Centre, Chris O’Brien Life House, Royal Prince Alfred Hospital, Sydney, Australia; 7 Hereditary Cancer Clinic, Prince of Wales Hospital, Sydney, Australia

**Keywords:** Implementation science, Spaced education, Practice change

## Abstract

Continuing medical education (CME) is challenging and often has limited impact on clinician behavior or patient outcomes. This study examined the impact of an online Qstream education program on senior clinicians to determine its utility for increasing clinician knowledge about the latest guidelines regarding genetic assessment and consideration of genetic testing for women with particular types of ovarian, fallopian tube and primary peritoneal cancer. Participants were recruited into a pilot study that involved responding to case-based scenarios at spaced and repeated intervals. At the completion of the program, semi-structured interviews were conducted to ascertain the impact on their knowledge and referral behavior. Findings from interviews were subject to thematic analysis that involved the identification of categories and themes. Twenty-one participants commenced the program, seventeen completed and twelve participated in semi-structured interviews. Thematic analysis yielded several themes including knowledge change, curriculum and format and changes in referral patterns. A majority of participants (*n* = 10) agreed the program had helped update their knowledge about referring women, and eight agreed they would now change their referral patterns. The use of QStream as an approach to CME has significant advantages when working with busy clinicians. QStream has a well accepted format and most participants indicated it is very appropriate for disseminating updates to clinical guidelines and protocols. It is important to supplement CME programs with other implementation techniques, such as audit and feedback as multifaceted approaches are more likely to result in behavior change.

## Introduction

Participation in continuing medical education (CME) is considered important for ensuring that doctors have access to evidence-based information that is designed to improve patient care. At the same time, the didactic format of most CME has been shown to be largely ineffective at changing clinician behaviour or improving patient outcomes [1]. Nevertheless, access to CME is crucial to enable doctors to keep up to date with rapidly evolving knowledge, including clinical guidelines. This is particularly relevant in oncology, where new research findings are constantly emerging and where new knowledge must be quickly translated into clinical practice to ensure treatment approaches are supported by the best available evidence.

Ovarian cancer is the eighth most common cause of cancer death worldwide [2]. This rate has not changed significantly over the past decade and it is estimated that one in 77 women in Australia will develop ovarian cancer by the age of 85 [3]. Women with a familial or genetic predisposition to ovarian cancer have a significantly increased risk of developing the disease, which warrants effective risk management strategies. It is now known that a significant proportion of the genetic risk of developing breast and ovarian cancer is due to germline mutations in the genes *BRCA1* and *BRCA2* [4]. In Australia, family cancer clinics (FCCs) offer a service for people with a family history of cancer and provide counselling regarding a person’s risk of developing cancer and, if appropriate, may also offer genetic testing [5]. This is important because risk-reducing strategies are effective for both breast and ovarian cancer, and early data suggest that in ovarian cancer, a *BRCA* mutation is associated with longer survival rates and generally favourable responses to platin-based chemotherapy [6].

n Australia, guidelines for predicting a person’s risk based Ion family history of breast and ovarian cancer have been developed [7], and in the state of New South Wales (NSW), criteria for determining who should be considered for BRCA 1/2 genetic testing were updated in 2012 [8]. Changes to the guidelines were based on new published data on the frequency of BRCA 1/2 mutations in the Australian population [6]. It is now recommended that women diagnosed with high-grade invasive ovarian, fallopian tube or primary peritoneal cancer (non-mucinous) and who are aged less than or equal to 70 years should be considered for testing [8]. In addition, testing should be considered for women with invasive ovarian, fallopian tube or primary peritoneal cancer (non-mucinous) at any age when there is a family history of breast or ovarian cancer [8].

One challenge in disseminating new knowledge is that clinical guidelines alone are not necessarily an effective means of knowledge translation [9], and, furthermore, the dissemination of new knowledge does not necessarily result in behaviour change in clinicians [10]. Hence, there is a need for innovative approaches to the dissemination of new knowledge and updated guidelines. This article reports on the development and implementation of an online QStream program that was used to disseminate updated clinical guidelines to clinicians about the referral of women with particular types of gynaecological cancer to FCCs.

The phase one study aimed to increase and/or reinforce clinician knowledge about the latest evidence regarding genetic assessment and consideration of genetic testing for women with particular types of ovarian, fallopian tube or primary peritoneal cancer. It also aimed to determine whether any knowledge acquisition reported by participants was associated with behaviour change, as reflected by an increase in the number of women subsequently referred for genetic assessment at two metropolitan hospital sites in Sydney. This pilot study, therefore, tests the viability of referral data collection methods needed to demonstrate behaviour change impacts and helps inform whether a wider dissemination of the program is warranted.

QStream (previously referred to as spaced education) was developed at Harvard Medical School and is delivered electronically by Internet or smart phone [11]. It takes advantage of the psychological finding that educational encounters which are spaced and repeated over time result in more efficient learning and improved learning retention [12, 13]. Several randomised studies have demonstrated its effectiveness in improving retention of learned material [14, 15], its positive impact on practice behaviours of health workers [16] and most importantly, patient outcomes [17]_._ Few studies, however, have examined whether behaviour change would result from a shorter intervention used to disseminate updates to clinical guidelines or tested behaviour change data collection methods.

Programs that use the QStream platform are built around participants answering a small number of case-based multiple choice questions that are emailed to them over a number of weeks. Upon answering a question, participants are given succinct feedback and links to further resources. In traditional QStream programs, participants are emailed a clinical case scenario with multiple choice questions [18]. Once the student has answered the question, they are provided with the correct answer, further explanations and links to other resources. The correct answer (or take-home message) is clearly and succinctly stated each time it is returned. The typical QStream program repeats each case scenario in a cycle ranging from 8 to 45 days [18]. In addition, most QStream programs have at least eight case scenarios but given the brief nature of the guideline changes, the current study relied on only three case scenarios highlighting key criteria for the referral of women.

Given that several studies have already demonstrated QStream’s efficacy in terms of learning and retention, this phase one study focused on identifying whether a brief QStream program would be acceptable and effective at disseminating updated clinical guidelines. The study also sought to identify whether a brief QSteam intervention would impact on participant behaviour and practice. In order to do this, pre- and post-data over a 12-month period on the number of women diagnosed with particular types of ovarian cancers and the number subsequently referred for assessment and consideration of BRCA 1/2 testing were collected. Hence, this study focuses on QStream’s effectiveness as a vehicle for translating new knowledge into clinical practice.

## Methods

A case-based QStream program was developed in collaboration with clinical investigators at the Sydney West Translational Cancer Research Centre (SW-TCRC) and Sydney Catalyst TCRC. The pilot study targeted clinicians involved in the care of women with ovarian cancer at two metropolitan hospitals in Sydney, Australia. A purposive sample of gynaecological and medical oncologists, along with their trainees was sent information about the study in October 2013. Key potential referrers of women at two sites were identified by clinical coinvestigators and were sent information about the study via email. Information and consent forms were also disseminated at regular oncology team meetings at both sites.

**Fig. 1:**
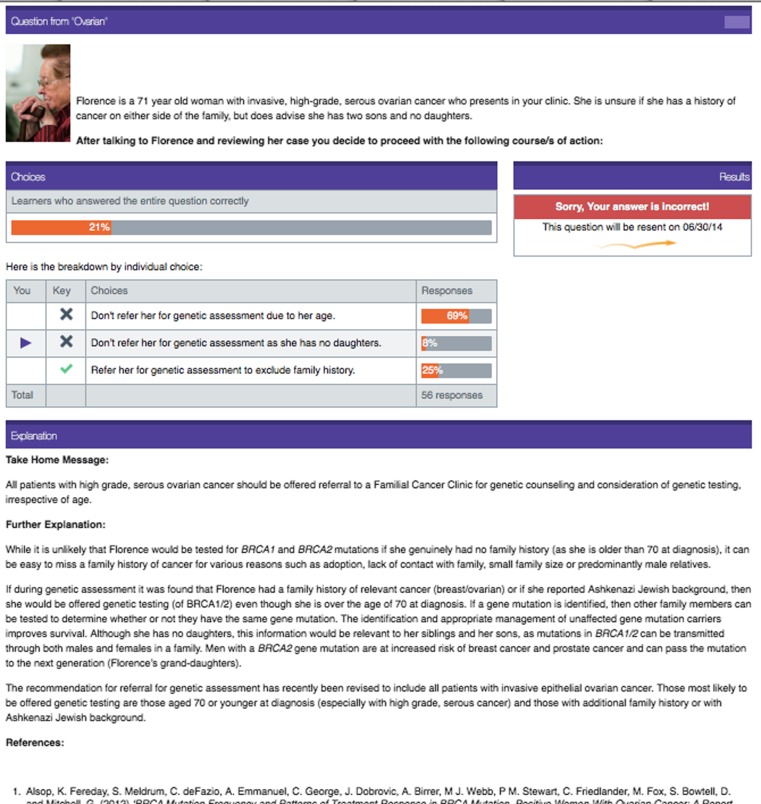
QStream case example

The final QStream program consisted of three case examples, for which participants had multiple choices of responses. Each case had one correct response and two incorrect options. If participants answered a question incorrectly, it was resent 5 days later. If the question was answered correctly it was resent 8 days later. Participants were required to answer each question correctly on two occasions before it was retired. The course was completed when all questions were retired. The cases and questions aimed to be relevant and challenging and were possible to mirror real clinical events. A case example from QStream is presented in Fig. 1.

The impact of the QStream education program was assessed by the use of semi-structured interviews that explored the program’s acceptability, participant knowledge and factors that enabled completion. Participants were invited to complete a telephone interview and were asked about their previous involvement in online education programs, how the QStream program impacted on their confidence and knowledge and whether they considered it would have any effect on their referral patterns for women diagnosed with invasive epithelial ovarian cancer, including primary peritoneal or fallopian tube cancer. All interviews were audio-taped and transcribed, and broad themes were identified.

In addition, quantitative data was collected to ascertain the impact of the program on referral rates by collecting data on the number of women referred for genetic assessment and/or testing by participants at both sites over a 12-month period pre- and post-QStream program. The number of women diagnosed with ovarian cancer, specifically the histological subtypes that are associated with BRCA 1/2 mutations in the pre- and post-twelve-month period at the two test sites was also determined. All referral data collected were assigned a number and de-identified and stored in compliance with the NSW Health Records and Information Privacy Act [19].

By using mixed methods, this study aims to explore the learning experience and knowledge acquisition of participants and, more importantly, whether this translates into behaviour change as reflected by the referral rates of women with particular types of ovarian cancer at two sites. This contributes to our understanding of the effectiveness of QStream when used as a brief intervention to disseminate updated clinical guidelines and whether it has wider applications and potential for dissemination. Approval was received from the Sydney Local Health District and the Western Sydney Local Health District Human Research Ethics Committees to conduct the study.

## Results

A total of 29 clinicians enrolled in the QStream online program and, of these, eight did not commence the course. Of the 21 participants who did commence the program, 18 (86 %) completed the program (8 from site A and 10 from site B). Eleven participants agreed to be interviewed at the completion of the program (five from Site A and six from Site B). Participants in the QStream program included five registrars, two nurses, four medical oncologists, four gynaecological oncology staff specialists, two gynaecological oncology fellows and one unstated. The final thematic analysis yielded several themes, including knowledge change, content and format (QStream as a learning platform) and perceived changes in referral patterns. The key themes and a range of exemplar quotes from participants are presented in Table 1.

**Table 1:** Themes and exemplar quotes

Themes	Exemplar quotations
Knowledge change	• There was a degree of clarification of my knowledge and some points that I wasn’t necessarily aware of • These things, were misconceptions that I had before that I have corrected with these answers • The information you guys gave in the QStream module is helping me bring up, you know, things with a little bit more confidence • I think it taught me a lot about, you know, who I should be sending patients to, or what else I should be thinking about. Do I need to think about genetics for this person or not? • I would have happily done more questions. It was such a good learning experience
Curriculum and format of QStream	• …the one question a day or whatever made it quite easy…the type of thing where you didn’t have to spend 20 min each time • It was short and to the point, and it didn’t require me to do five thousand screen changes and bits and pieces to physically do it. Hmm. It was short, sharp and to the point • I think the, the fact that it was organized and that it came up on my iphone, um, actually was, was very good…it fitted very well with my schedule) • I don’t have time or energy to sit down for half an hour or forty five minutes to click through a million screens to get to the point. If it’s one of those ten minute things, or five minute things like the Qstream was, then I’d be more than interested • Obviously, people don’t necessarily have a long attention span for multiple pages worth of stuff and so because it was relatively short, sharp and shiny and got to the point that made it quite easy • It was really good to be honest. And because it comes in small amounts, like, you can do five or ten minutes of, you know, after a twelve hour day, you can still do that • It doesn’t take much time and you do learn a fair bit quite quickly • I think it’s a great way because it’s case based and there’s a couple of questions attached to each case and then the answer is just a couple of paragraphs explaining, you know, the correct way of approach for managing specific cases
Dissemination of updated clinical guidelines	• …I think it might be quite a good way of doing, of um, disseminating that change in guidelines …I found it was quite helpful but I guess the logistics as to would have to be reconsidered for Qstream • If it can be succinct then it is easier to do via Qstream • Medscape is giving you news, Qstream is testing your knowledge of a particular thing. There is a different thing all together • At the moment we have easy access to our consultants and we could easily ring up the genetic counsellors and ask them whether it’s an appropriate referral . If I’m not sure I’d just ring them up and ask them
Changes in referral patterns	• I only just started learning about gynaecological cancer, so I didn’t know very much about who you would refer and who you wouldn’t refer. But based on that I think it taught me a, a lot about, you know, who I should be sending patients to, or what else I should be thinking about. Do I need to think about genetics for this person or not • Whereas with ovarian cancer what I’ve obviously realised is it’s [referral] not related to their age and it is about family history and a lot about their pathology • Yes, it’s probably going to. Yes, because there are a lot of times, as I said I understand that we need to send more people, more women to genetic testing • I think at least it’s given me enough confidence to know whether to refer someone or not

### Knowledge Change

A majority of participants agreed that the program had helped them update their knowledge about referring women for assessment and consideration of genetic testing. Ten participants agreed that the program increased their knowledge and, as one participant noted, ‘*a lot of the time we don’t actually learn why you would refer someone for genetics…so that was good and there were several genetic things that I thought that we shouldn’t do and it turned out that we should do*’. Furthermore, even when the program was not seen as changing participant knowledge, its importance for disseminating new knowledge to colleagues was acknowledged; ‘*Interestingly enough, one of my colleagues was not aware of the new recommendations for screening…so it kind of prompted an interesting offline conversation about that and we went and looked up an article that supported that recommendation*’. In addition, comments were received from participants that suggest the information contained in case studies was retained; ‘*More than anything it’s just a good learning tool …And because you’re answering the question, you’re actually remembering it more than if you were just reading*’.

### QStream as a Learning Platform

The curriculum and format of the QStream program were well received. None of those interviewed had prior experience of QStream as a learning platform and, overall, participants welcomed the fact that they could access the program on their phones and enjoyed the fact that it took little time to complete, *‘It fitted very well with my schedule. I think it can sometimes be tough to sort of take the time to sit down at a desk at home to do work. This actually worked well*’. The QStream format of multiple choice questions with links to further information was also well received, as was the fact that questions were emailed to participants, providing them with prompt reminders and easy access; ‘*The email is much easier to access and much easier to remember to open, rather than going to a different site and open the website and answer the question. So the way that the questions were sent to the email was very convenient*’. Eight of the 11 participants (73 %) interviewed affirmed that QStream is appropriate for disseminating new or updated clinical guidelines. ‘*I think it could work, …I think what was nice about it was the fact that it was fairly digestible, it wasn’t overwhelming*’. In contrast, two participants expressed that QStream would not be suitable for disseminating new or updated guidelines either because they did not see the question, answer format as suitable or else because they already have contact with genetic counsellors who they access prior to making any referrals; ‘*So [we get] all the updates …I don’t know necessarily if I would need to go through a question answer session*…’.

Although most participants enjoyed the short and sharp format of QStream, several participants stated they would have liked more information and more of an educational component in future programs, ‘*I got all the questions right and I was hoping to do some more stuff and learn a little bit more…I would have liked to do a little bit more*’.

### Practice Change

Eight participants agreed that the program had changed the way they would now refer women for genetic counselling and consideration of testing, ‘*absolutely …at the back of my mind I will know when and who needs it*’. Another participant noted that ‘*I would now refer people at a much lower threshold than I would before*’. Participants also appeared to understand the need for counselling, if not for genetic testing, ‘*One it’s the age difference but two it’s the opportunity, even if people don’t quite fit the criteria … any person who has had a diagnosis of high grade ovarian cancer, to have an opportunity to speak to a genetic counsellor even if they don’t end up getting full genetic testing*’.

The number of referrals to an FCC was determined at both test sites in a 12-month period following the QStream program and compared with a 12-month period prior to the program. Overall, across the two sites, the number of women diagnosed with ovarian cancer that would be eligible for referral to an FCC in the two-time period was similar (97 pre and 110 post), as would be expected; however, there was a 1.8-fold increase in referrals at site A (26 referrals pre and 47 referrals post) and a 1.6-fold increase at site B (28 referrals pre and 46 referrals post).

## Discussion

This study demonstrates that the use of QStream as an approach to teaching and learning has significant advantages when working with busy clinicians. QStream has a well-accepted format and most participants indicated that it is very appropriate for disseminating updates to clinical guidelines. In addition, the referral data supports that the new knowledge acquired by participants did translate into practice change as reflected by the increase in referral rates at both sites post-intervention. This is important given that this pilot study was designed to help inform whether a wider dissemination of the QStream program among oncologists and surgeons across Australia is warranted.

Several limitations to the current study must be noted. Firstly, given the specialised nature of the subject matter, the small number of participants was a limitation and did not allow formal statistical analysis. It was, however, adequate for a pilot study to test the impact of the education program on the significance and knowledge change among a small and specialised group of clinicians. Another limitation is that the findings relating to knowledge change were gleaned from qualitative interviews, largely because of the brief nature of the take-home messages in the program and because a pre-course knowledge test may have acted as a confounding factor in participant knowledge acquisition. A randomised controlled trial is now needed to confirm that improvements in participant knowledge are a result of the QStream program and to explore the extent of increased awareness that occurs in the absence of an education intervention.

The literature is replete with examples of how difficult it can be to translate new research findings into changes in clinical practice and patient outcomes [10, 17]. Although the referral data in the current study is encouraging, it needs to be interpreted with caution. Other factors may have contributed to the increased referrals in addition to the QStream program. We have shown that the increase was not due to an increase in the number of women diagnosed with ovarian cancer. Furthermore, this phase one study contributes to our understanding of the data collection methods that education researchers need to demonstrate behaviour change outcomes. These behaviour change outcomes are crucial for larger implementation studies that aim to fully explore the impact of medical education programs. Hence, this phase 1 data will be used to power a randomised controlled study to confirm the impact of the methodology on practice.

The fact that differing methods are used to collect and record cancer incidence data across sites in one metropolitan centre highlights how, despite the widespread use of implementation approaches, considerable hurdles remain in being able to demonstrate that what is learned in the research setting is translated into clinical practice. Given that there is broad acceptance that multifaceted interventions are required to ensure the timely translation of new research findings [20], it may be necessary to supplement QStream programs with other implementation techniques, such as audit and feedback to ensure behavioural change is present and persists beyond the implementation phase.

Although there was a 13 % attrition rate (for those who commenced the course), it is consistent with attrition rates reported by other online education programs [21]. Given that the pilot program targeted senior clinicians and involved no formal CME points, the completion rate was considered acceptable for this particular cohort. Overall, this study demonstrates that the QStream platform is one mechanism for disseminating new research or guidelines among clinicians in high work volume environments. Further implementation and knowledge translation studies are needed to identify appropriate data collection methods that capture behaviour change in diverse clinical settings.

Because ovarian cancer is one of the most serious cancers for women and symptoms can be hard to detect, it is vital to prevent this disease in those at high risk. For this reason, it is important that effective mechanisms for disseminating new research findings are adopted because if mutations in *BRCA1* or *BRCA2* can be identified and at-risk relatives are able to adopt preventive measures, the incidence of ovarian cancer may be reduced. This study did address an important gap in knowledge translation and despite being unable to randomise participants, the findings do support that QStream has utility for disseminating new research findings and updated clinical guidelines.
